# Valethamate bromide: Conflicting evidence and continuing use

**DOI:** 10.4103/0976-500X.72365

**Published:** 2010

**Authors:** Thirunavukkarasu Arun Babu, Vijayan Sharmila

**Affiliations:** *Department of Pediatrics, Sri Lakshmi Narayana Institute of Medical Sciences (SLIMS), Osudu, Agaram Village, Puducherry, India*; 1 *Department of Obstetrics and Gynecology, Indira Gandhi Medical College and Research Institute (IGMC&RI), Puducherry, India*

Dear Editor,

We read the editorial ‘Valethamate bromide: Is there any proof of efficacy and safety for its use in labour?’ by author Gitanjali B, with interest.[1] The editorial excellently describes the inadequate literature and unconvincing evidence regarding the use of valethamate bromide for cervical ripening and dilatation in labor. However, with due respect to the author, we would like to raise certain points. Firstly, we feel that the article is more biased and judgmental rather than being open to all currently available evidence. Contradictory to what is stated in the article, many clinical trials have shown that valethamate bromide is effective in facilitating cervical ripening, dilatation and thereby decreasing the duration of labor.[2–5] But, the effect is not seen consistently and two trials have found no significant effect with the use of this drug.[6,7]

We also wish to differ regarding the first of the three reasons suggested by the author for using this drug, which states ‘that it is beneficial in crowded labor rooms to facilitate the reduction in time spent monitoring patient rather than a sound medical reason’. The most important reason for using this drug is to facilitate the labor process and reducing labor duration with an ultimate aim of reducing fetomaternal complications secondary to delayed or nonprogressive labor.

It is also important to remember that this drug has been used for almost three decades now and most of the clinicians have a good experience with this drug. Although few adverse effects like self-limiting maternal tachycardia can occur, which can be easily managed in low-risk patients, no major life-threatening adverse reactions have been reported till date. This should not prevent us from using this drug because the benefit is more than the posed risk. So we feel the debate whether or not to use this drug is still wide open and the current evidence does not warrant stoppage of using this drug. Large-scale multicentric randomized controlled clinical trials are needed before any major conclusions can be drawn in this regard.
